# Congenital heart disease in 22q11.2 deletion syndrome: a meta-analysis and systematic review of the literature

**DOI:** 10.1136/jmg-2025-110624

**Published:** 2025-07-31

**Authors:** Carina Sauter, Michael Hofbeck, Paula Franz, Laura Kettenstock, Klara Steger, Matthias Linhardt, Andrea Reiter, Stefan Störk, Marcel Romanos, Franziska Radtke

**Affiliations:** 1 University Hospital Würzburg, Center of Mental Health, Department of Child and Adolescent Psychiatry, Psychosomatics and Psychotherapy, Würzburg, Germany; 2 University Hospital Tübingen, Department of Pediatric Cardiology and Intensive Care Medicine, Tübingen, Germany; 3 German Center of Prevention Research in Mental Health, Würzburg, Germany; 4 Institute of Psychology, Julius Maximilians University Würzburg, Würzburg, Germany; 5 University Hospital Würzburg, Department Clinical Research & Epidemiology, Comprehensive Heart Failure Center, Würzburg, Germany; 6 University Hospital Würzburg, Department of Internal Medicine I, Würzburg, Germany

**Keywords:** Heart Defects, Congenital, Cardiovascular Abnormalities, Genetic Testing

## Abstract

The 22q11.2 deletion syndrome (22q11.2DS) results from a heterozygous deletion at chromosomal locus 22q11.2 and is associated with multisystem symptoms, including cardiovascular, psychiatric and palatal manifestations. Although congenital cardiovascular aberrations are frequent in patients with 22q11.DS, exact prevalence figures remain unclear. Literature was searched in August 2022 and updated in May 2024 using the databases PubMed, Web of Science and Cochrane library to retrieve studies in English and German focusing only on studies involving 22q11.2DS. Prevalence data for cardiovascular aberrations were arcsine transformed and summarised by random-effects models using Meta-XL. Evidence quality was assessed via the Grading of Recommendations, Assessment, Development and Evaluation (GRADE) approach. The systematic searches identified 4338 studies, of which 7 were included for the meta-analysis of prevalence using random-effects models and sensitivity analyses. The pooled prevalence for heart defects (mean; 95% CI) was found to be elevated for tetralogy of Fallot (20%; 0.17 to 0.23), ventricular septal defect (14%; 0.12 to 0.16), pulmonary atresia with ventricular septal defect (9%; 0.06; 0.12), interrupted aortic arch (10%; 0.06 to 0.15), truncus arteriosus communis (9%; 0.07 to 0.12) and atrial septal defect (3%; 0.01 to 0.04). The risk of bias of the included studies was low to moderate. This study, to our knowledge, represents the first systematic review and meta-analysis of prevalences for congenital cardiovascular aberrations in individuals with 22q11.2DS. The high frequencies observed underline the need for cardiovascular screening in patients with 22q11.2DS and genetic screening for 22q11.2DS in congenital heart disease.

## Introduction

 The 22q11.2 deletion syndrome (22q11.2DS) results from a heterozygous deletion at chromosomal locus 22q11.2 and is the most prevalent microdeletion syndrome.^1^ It is associated with various comorbidities, including palatal anomalies, immune deficiency and psychiatric symptoms.^2 3^ Additionally, cardiac anomalies are frequently present in these patients. Indeed, 22q11.2DS has been suggested as the second main cause of congenital heart diseases (CHDs).^4^ CHD has a huge impact on individuals’ quality of life and mortality rates. It has been determined that cardiac defects are the primary cause of mortality in children with 22q11.2DS.^5^ Additionally, sudden death and heart failure are frequent causes of mortality among adults with 22q11.2DS.^6^ In adults with non-syndromal CHD, the mortality rate is 16-fold higher compared with healthy controls.^7^ Furthermore, adults with CHD achieved lower education and were significantly more often unemployed.^8^

Numerous CHDs are commonly reported in the literature pertaining to the 22q11.2DS population, including tetralogy of Fallot (TOF), pulmonary atresia with ventricular septal defect (PA+VSD), truncus arteriosus communis (TAC), ventricular septal defect (VSD), atrial septal defect (ASD), interrupted aortic arch (IAA) and aortic arch anomalies (AAA).^9^ However, specific prevalence numbers regarding cardiovascular abnormalities in 22q11.2DS remain understudied. A systematic review and meta-analysis of 260 studies was conducted to define the global birth prevalence of CHD for the period between 1970 and 2017 and reported a mean prevalence of 0.8%.^10^ However, there was no focus on prevalence numbers of CHD in individuals with 22q11.2DS. Concerning 22q11.2DS, several literature reviews have been undertaken to display prevalence figures of CHD.^5 11 12^ Nevertheless, the results of these reviews were not obtained systematically and were subject to selection bias, since many data were collected at specialised cardiology centres representing a preselection of individuals with cardiovascular anomalies. Therefore, the occurrence of CHD may be overestimated and may not accurately reflect the actual prevalence of CHD in 22q11.2DS.

Currently, no systematic review and meta-analysis has examined this topic; therefore, this study aims to fill this gap by assessing the prevalence rates of CHD in 22q11.2DS.

## Methods

The Preferred Reporting Items for Systematic reviews and Meta-analyses (PRISMA) were used for this study.^13^

### Search and selection strategies

A systematic search was performed in the databases PubMed, Cochrane Library and Web of Science on 18 August 2022 and updated on 22 May 2024 using synonyms of 22q11.2DS AND cardiovascular diseases. For complete search strings, see online supplemental file. Search results were reviewed by author CS based on title and abstract and reviewed by author FR. CS retrieved the full texts and ambiguities were discussed with FR.

We included studies (1) concentrating on a sample comprising of N>35; (2) diagnosing 22q11.2DS through methods such as fluorescence in-situ hybridisation (FISH), multiplex ligation-dependent probe amplification (MLPA), quantitative fluorescent PCR or microarray; and (3) providing raw data or prevalence figures (%) of CHD. The following exclusion criteria were applied: (1) studies not written in English or German; (2) studies with no focus on 22q11.2DS; (3) systematic and non-systematic reviews, conference papers, study protocols, case series or reports, animal studies; (4) studies where patients were recruited from a cardiological department/hospital; (5) studies that did not report methodology for diagnosing CHD.

### Outcome measure

The main objective was to determine the prevalence of CHD in patients with 22q11.2DS. The relevant heart defects were: TOF, PA+VSD, TAC, IAA, VSD, ASD and AAA (including anomalies of laterality and branching of the aortic arch like right aortic arch, aberrant subclavian artery, double aortic arch and vascular rings).

### Quality assessment

Risk of bias (RoB) was evaluated using 10 questions developed by Hoy *et al* for prevalence studies addressing selection bias, non-response bias, measurement bias and bias related to the analysis.^14^ Studies were scored between 0 and 10. One point was awarded for each ‘Yes’ answer in case no bias was found. Accordingly, 0–3 points indicated high RoB, 4–7 points moderate RoB, and 8–10 points low RoB. The Grading of Recommendations, Assessment, Development and Evaluation (GRADE) approach^15^ was applied. According to GRADE, a summary of quality of evidence was given for each outcome, highlighting aspects such as RoB, inconsistency, indirectness, lack of precision and publication bias.

### Data synthesis

A priori a threshold of N=35 for identifying studies with a sufficiently large sample size was used to define coverage probabilities of 95% Clopper-Pearson CIs with a width of 35% that are used for binomial proportion differences.^16 17^ The Clopper-Pearson CI was calculated as follows:


$$CI_{R}=\left[Beta_{\alpha /2}((E,N-E+1)\cdot 100.000,Beta_{1-\frac{\alpha }{2}}(E+1,N-E)\cdot 100.000)\right]$$

where *E* represents the observed events, *N* the average population and *Beta*_α_ (*p,q*) the 100α percentile of the beta distribution with the parameters *p* and *q*.^18^

The goal of our analysis was to estimate the pooled prevalence of each subtype of CHD. Prevalence is defined as the proportion of cases divided by the population size at the time of the survey.^19^ To calculate the pooled prevalences, we used random-effects models (DerSimonian & Laird)^20^ and applied the double arcsine transformation for the proportions and its CI, as recommended to be preferred over the logit transformation,^19^ using Meta-XL.^20^ We conducted an exact Fisher-Freeman-Halton test (two-sided) to define non-heterogeneous groups, using IBM SPSS Statistics Version: 29.0.0.0 (241) and a significance level smaller than 0.05.^21^ If studies were deemed significantly heterogeneous regarding a specific CHD, sensitivity analyses were conducted with studies N≥100 to account for a small study bias. Second, if studies were still deemed significantly heterogeneous, outliers were identified by leave-one-out analysis in Meta-XL. It estimates the same meta-analysis several times, based on the full set of identified studies minus one, respectively, and a study is regarded as an outlier if its exclusion results in non-heterogeneous groups per the p value of Cochran’s Q (Meta-XL) and exact Fisher-Freeman-Halton test (SPSS). I² estimates and Cochran’s Q were provided to describe heterogeneity.

## Results

### Characteristics of the included studies

**Figure 1:**
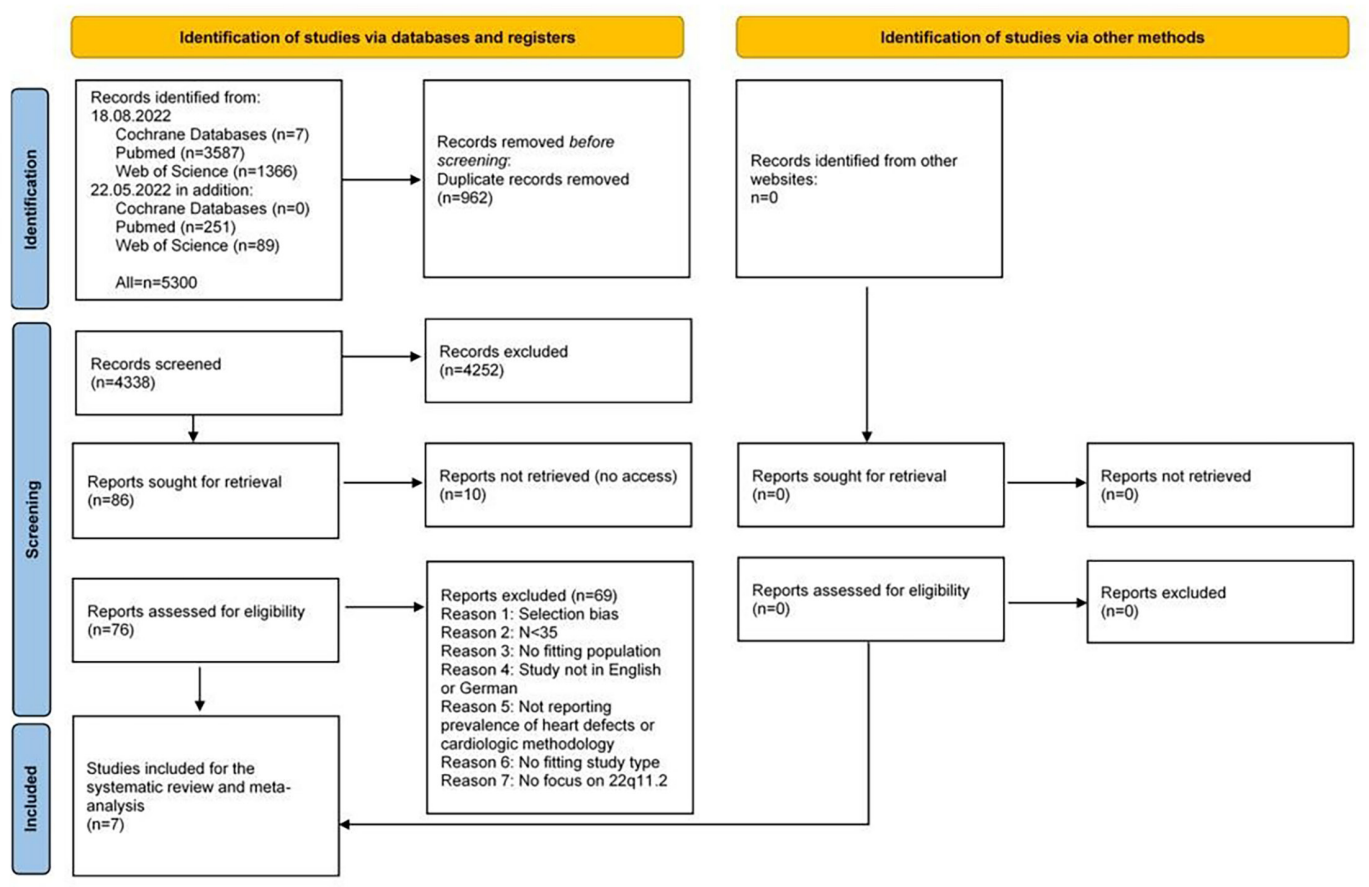
PRISMA (Preferred Reporting Items for Systematic reviews and Meta-analyses) flowchart showing the systematic literature search. 5300 studies were found in the systematic literature search, of which 4338 were screened for title and abstract after removal of duplicates. Of those, 4252 were excluded and 86 selected for full-text screening. Ten studies could not be retrieved because of no access; hence, 76 studies were assessed for eligibility and screened in full text. Further, 69 studies were excluded and 7 studies were included for the systematic review and meta-analysis.

The systematic literature search identified 4338 abstracts published from 1959 to 2024. Of the 76 articles screened in full text, 7 studies were included^22^,^28^ (PRISMA flowchart, figure 1). The included studies were retrospective, cross-sectional or longitudinal single-centre studies conducted in different countries worldwide. A total of 2519 patients with 22q11.2DS were involved. In all studies, 22q11.2DS was confirmed genetically either by FISH (7 studies), MLPA (3 studies), single nucleotide polymorphism-/comparative genomic hybridization array (SNP-/CGH-array) (1 study) or microsatellite polymorphisms (1 study). Diagnoses of CHD were verified through cardiology reports, medical records, echocardiography, cardiac catheterisations and/or MRI. One study compared interview data with medical records of patients to determine heart defect prevalence. Another study incorporated data from 23 European centres collected by questionnaires asking for data on heart, palate, renal and thymus anomalies, parathyroid function, growth, developmental status, behaviour and psychiatric illness. Characteristics of the included studies are shown in table 1 and in the online supplemental file.

**Table 1:** Overview of characteristics of the included studies

Study	Study type, country	Sample size, population, age and gender	Selection (inclusion, recruitment)	Genetical confirmation	Diagnostic method for outcomes	Outcomes diagnosed
Guo *et al*, 2017^23^	Retrospective study, USA	n=1472 with 22q11.2DS Age: not reported 49% male, 51% female	Recruitment by the International Chromosome 22q11.2DS consortium, the International 22q11.2 Brain Behaviour Consortium and clinical groups that specialise in the treatment of individuals with 22q11.2DS	FISH or MLPA	Cardiology reports, echocardiography	TOF
Grassi *et al*, 2015^22^	Cross-sectional study, Brazil	n=60 with 22q11.2DS Age: average: 4.8 years, range from 14 days to 20 years 57% male, 43% female	Patients followed at the Allergy and Immunology Unit and Genetic Unit at Instituto da Criança of HC-FMUSP; some were referred from the Paediatric Cardiology Unit of Instituto do Coração (INCOR)–HC-FMUSP	FISH and/or MLPA	Clinical and imaging evaluations	TOF VSD IAA TAC ASD
Lima *et al*, 2010^24^	Cross-sectional study, Norway	n=60 with 22q11.2DS Age: range from 1 to 54 years 47% male, 53% female	Recruitment through the genetic institutions in Norway	FISH	Interview and medical records of patients, reports from echocardiography, cardiac catheterisation and radiographs	TOF VSD IAA+VSD PA+VSD ASD
Repetto *et al*, 2009^27^	Retrospective study, Chile	n=208 with 22q11.2DS Age: mean: 5.2 years (of 182 patients); range from newborn to 39 years 49% male, 51% female	Recruited from five medical centres	FISH	Medical records, information based on electrocardiography, cardiac catheterisation or MRI	TOF VSD IAA Type B TAC ASD Other (such as AAA)
Óskarsdóttir *et al*, 2005^25^	Cross-sectional study, Sweden	n=100 with 22q11.2DS Age: range from 0.01 to 19.4 years 46% male, 54% female	First 100 children and adolescents younger than 20 years, admitted to the Queen Silvia Children’s Hospital in Göteborg, children were referred from all over Sweden	FISH	Echocardiography and/or cardiac catheterisation, findings from cardiac surgery	TOF VSD IAA Type B TAC PA+VSD ASD
Ryan *et al*, 1997^28^	Retrospective study, UK	n=545 with 22q11.2DS Age: range from newborn to 51 years old 49% male, 51% female	Data recruited from 23 European centres	FISH or microsatellite polymorphisms	Data from 23 European centres collected by questionnaires asking for data on heart, palate, renal and thymus anomalies, parathyroid function, growth, developmental status, behaviour and psychiatric illness	TOF VSD IAA PA+VSD TAC ASD AAA
Putotto *et al*, 2022^26^	Longitudinal single-centre study, Italy	n=74 with 22q11.2DS Age: mean: 27.5 years ≥16 years 59.5% male, 40.5% female	Department of Maternal Infantile and Urological Sciences of Sapienza University of Rome	SNP-/CGH-array, MLPA or FISH	Transthoracic echocardiogram	AAA

AAA, aortic arch anomalies; ASD, atrial septal defect; CGH, comparative genomic hybridization; FISH, fluorescence in-situ hybridisation; IAA, interrupted aortic arch; IAA+VSD, interrupted aortic arch with ventricular septal defect; MLPA, multiplex ligation-dependent probe amplification; PA+VSD, pulmonary atresia with ventricular septal defect; 22q11.2DS, 22q11.2 deletion syndrome; SNP, single nucleotide polymorphism; TAC, truncus arteriosus communis; TOF, tetralogy of Fallot; VSD, ventricular septal defect.

### Quality assessment

The application of the quality assessment by Hoy *et al*^14^ for prevalence studies revealed low to moderate RoB in all studies. Non-response bias was the most frequent type of bias (43%), followed by measurement bias (36%). Selection bias was observed in 19%, while no bias related to the analysis (0%) was found. Application of the GRADE approach led to a low quality of evidence (⊕⊕) for TOF, PA+VSD, TAC, VSD and ASD, and to a very low quality of evidence (⊕) for IAA because of inconsistency and for AAA because of lack of precision.

### Prevalences of CHD in 22q11.2DS

#### Ventricular septal defect (VSD)

**Figure 2:**
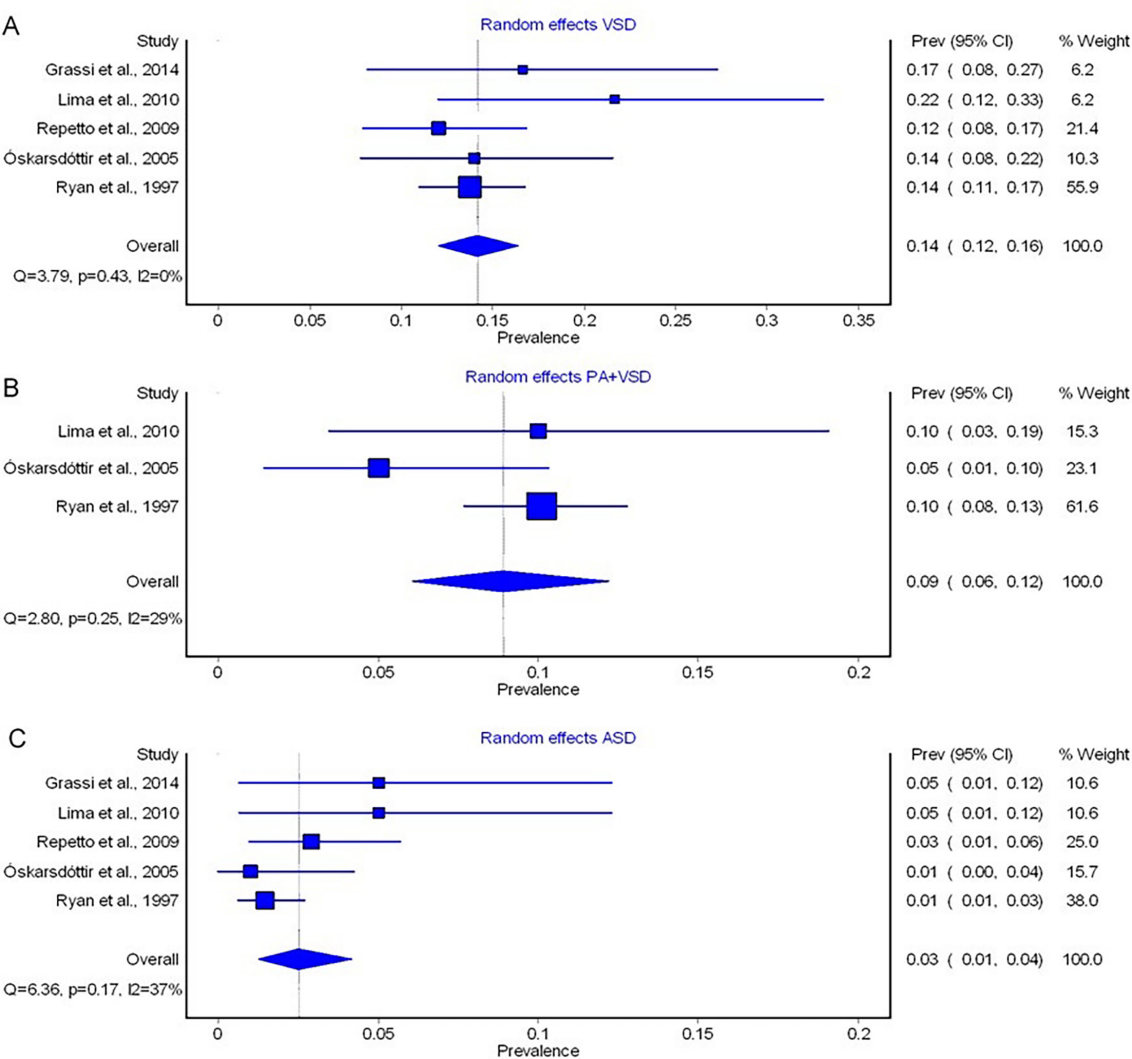
Forest plot of the prevalences of ventricular septal defect (VSD) (A), pulmonary atresia with ventricular septal defect (PA+VSD) (B) and atrial septal defect (ASD) (C) calculated by random-effects models in MetaXL. The included studies are listed on the left side, prevalence rates of each study with 95% CIs are shown on the first column on the right side. The second column on the right side represents the weight a study represents on the meta-analytically calculated pooled prevalence. The diamond on the bottom represents the pooled prevalence. I^2^, percentage of variation in prevalence across studies that is due to heterogeneity rather than chance; p, significance level of Q derived by χ² distribution; Q, Cochran’s Q.

VSD was observed in individuals with 22q11.2DS across five studies,^22 24 25 27 28^ with no significant evidence indicating heterogeneity (p=0.401) according to the exact Fisher-Freeman-Halton test. The pooled prevalence of VSD was 14% (95% CI 0.12%; 0.16%), and there was no heterogeneity detected (I²=0%, Q=3.79, p=0.43) (figure 2A).

#### Pulmonary atresia with ventricular septal defect (PA+VSD)

Three studies reported on the prevalence of PA+VSD.^24 25 28^ According to the exact Fisher-Freeman-Halton test, we found no significant evidence for heterogeneous groups (p=0.287). The pooled prevalence for PA+VSD was found to be 9% (95% CI 0.06%; 0.12%), with I² indicating no serious heterogeneity (I²=29%, Q=2.8, p=0.25)^29^ (figure 2B).

#### Atrial septal defect (ASD)

Five studies reported the prevalence of ASD in 22q11.2DS.^22 24 25 27 28^ The exact Fisher-Freeman-Halton test did not reveal significantly heterogeneous groups (p=0.060). The random effects models revealed a pooled prevalence of 3% (95% CI 0.01%; 0.04%), with I² indicating moderate heterogeneity (I²=37%, Q=6.36, p=0.17)^29^ (figure 2C).

#### Aortic arch anomalies (AAA)

Three studies reported the prevalence of isolated AAA. AAA accounted for 12 out of 545 cases (5 patients with right aortic arch, 5 with aberrant subclavian artery, 2 with double aortic arch) in the study by Ryan *et al*, resulting in a prevalence of 2.2% (95% CI 0.01%; 0.04%) in 22q11.2DS.^28^ Repetto *et al* grouped AAA (aberrant subclavian artery, right-sided aortic arch, vascular ring and double aortic arch) under ‘other anomalies’ and reported a frequency of 27 out of 208 patients (13%). However, as the group ‘other anomalies’ also included patent ductus arteriosus, no exact prevalence could be derived.^27^ Putotto *et al* reported a prevalence of 27% for AAA including double aortic arch (n=1), right aortic arch (n=15), right and left aortic arch with aberrant left subclavian artery (n=8, n=5) and vascular ring (n=2).^26^ Because of the limited number of studies and no clear distinction of the outcomes, no meta-analytical calculation was conducted.

#### Tetralogy of Fallot (TOF)

**Figure 3:**
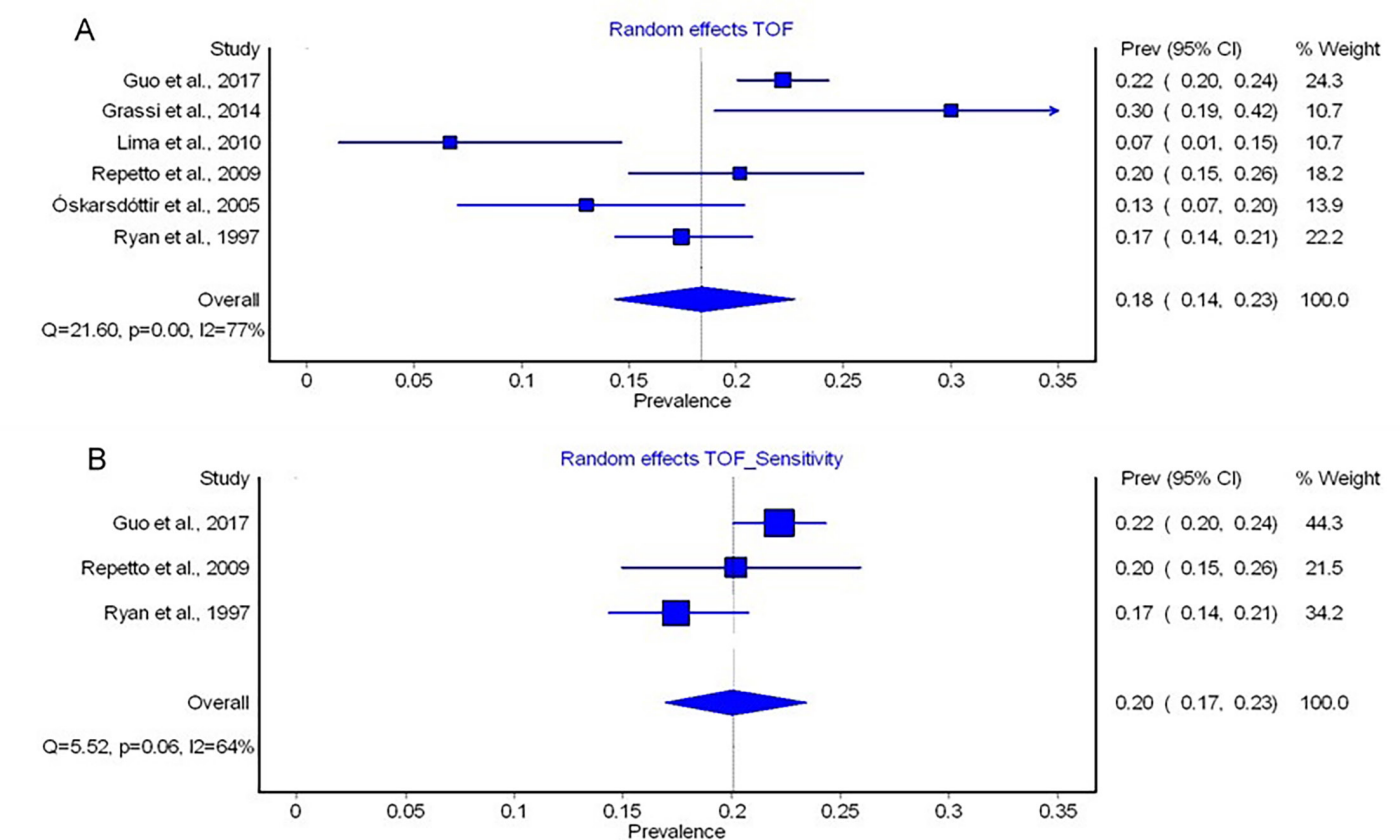
Forest plot of the prevalences of tetralogy of Fallot (TOF) calculated by use of random-effects models in MetaXL (A) and forest plot of the prevalences of TOF calculated by sensitivity analysis by use of random-effects models in MetaXL (B). The included studies are listed on the left side, prevalence rates of each study with 95% CIs are shown on the first column on the right side. The second column on the right side represents the weight a study represents on the meta-analytically calculated pooled prevalence. The diamond on the bottom represents the pooled prevalence. I^2^, percentage of variation in prevalence across studies that is due to heterogeneity rather than chance; p, significance level of Q derived by χ² distribution (df=2); Q, Cochran’s Q.

TOF was reported in six studies.^22^,^2527 28^ The exact Fisher-Freeman-Halton test indicated heterogeneity (p<0.001). A pooled prevalence of 18% (95% CI 0.14%; 0.23%) for TOF was found when all studies were included (I²=77%, Q=21.60, p=0.09) (figure 3A). Due to significant heterogeneity, we further conducted a sensitivity analysis by including a subset of k=3 studies with a sample size >100. The meta-analysis on this subset of larger (and better-powered) studies indicated no significantly heterogeneous groups (exact Fisher-Freeman-Halton test p=0.065).^23 27 28^ Comparable to the analysis covering all studies, this follow-up analysis found a pooled prevalence of 20% (95% CI 0.17%; 0.23%) for TOF. I² indicated substantial heterogeneity (I²=64%, Q=5.52, p=0.06)^29^ (figure 3B).

#### Truncus arteriosus communis (TAC)

**Figure 4:**
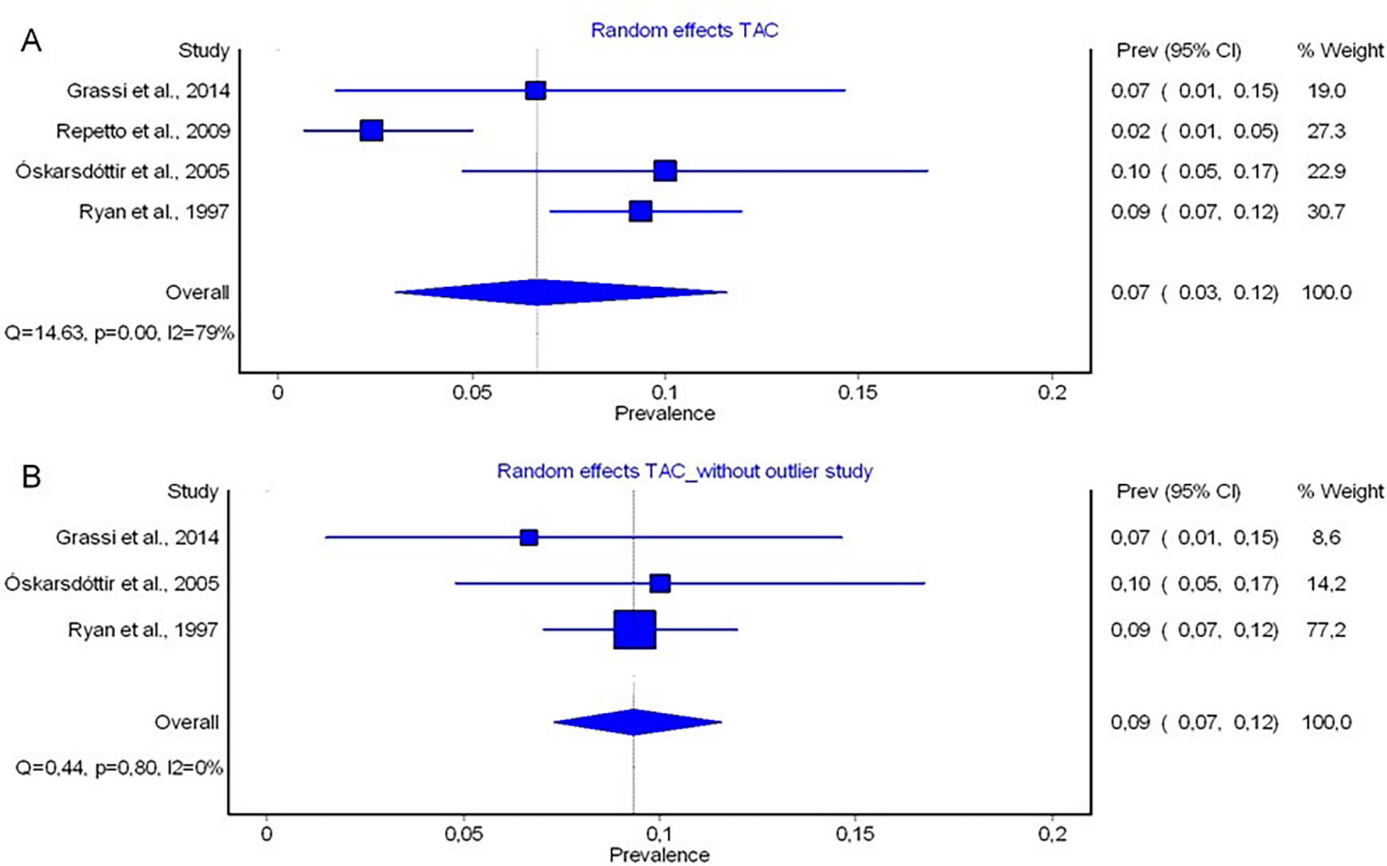
Forest plot of the prevalences of truncus arteriosus communis (TAC) calculated by random-effects models in MetaXL (A) and forest plot of the prevalences of TAC calculated by leave-one-out analysis by use of random-effects models in MetaXL (B). The included studies are listed on the left side, prevalence rates of each study with 95% CIs are shown on the first column on the right side. The second column on the right side represents the weight a study represents on the meta-analytically calculated pooled prevalence. The diamond on the bottom represents the pooled prevalence. I^2^, percentage of variation in prevalence across studies that is due to heterogeneity rather than chance; p, significance level of Q derived by χ² distribution (df=2); Q, Cochran’s Q.

In four studies, prevalence of TAC was reported.^22 25 27 28^ The studies showed significant heterogeneity (exact Fisher-Freeman-Halton test p=0.004). Including all four studies in the meta-analysis, a pooled prevalence of 7% (95% CI 0.03%; 0.12%) was calculated (I²=79%, Q=14.63, p=0.00) (figure 4A). We identified one study^27^ as an outlier using leave-one-out analysis. After exclusion of Repetto *et al*,^27^ exact Fisher-Freeman-Halton test (p=0.823) indicated no significant heterogeneity and the pooled prevalence for TAC was 9% (95% CI 0.07%; 0.12%) with no heterogeneity (I²=0%, Q=0.44, p=0.80) (figure 4B).

#### Interrupted aortic arch (IAA)

**Figure 5:**
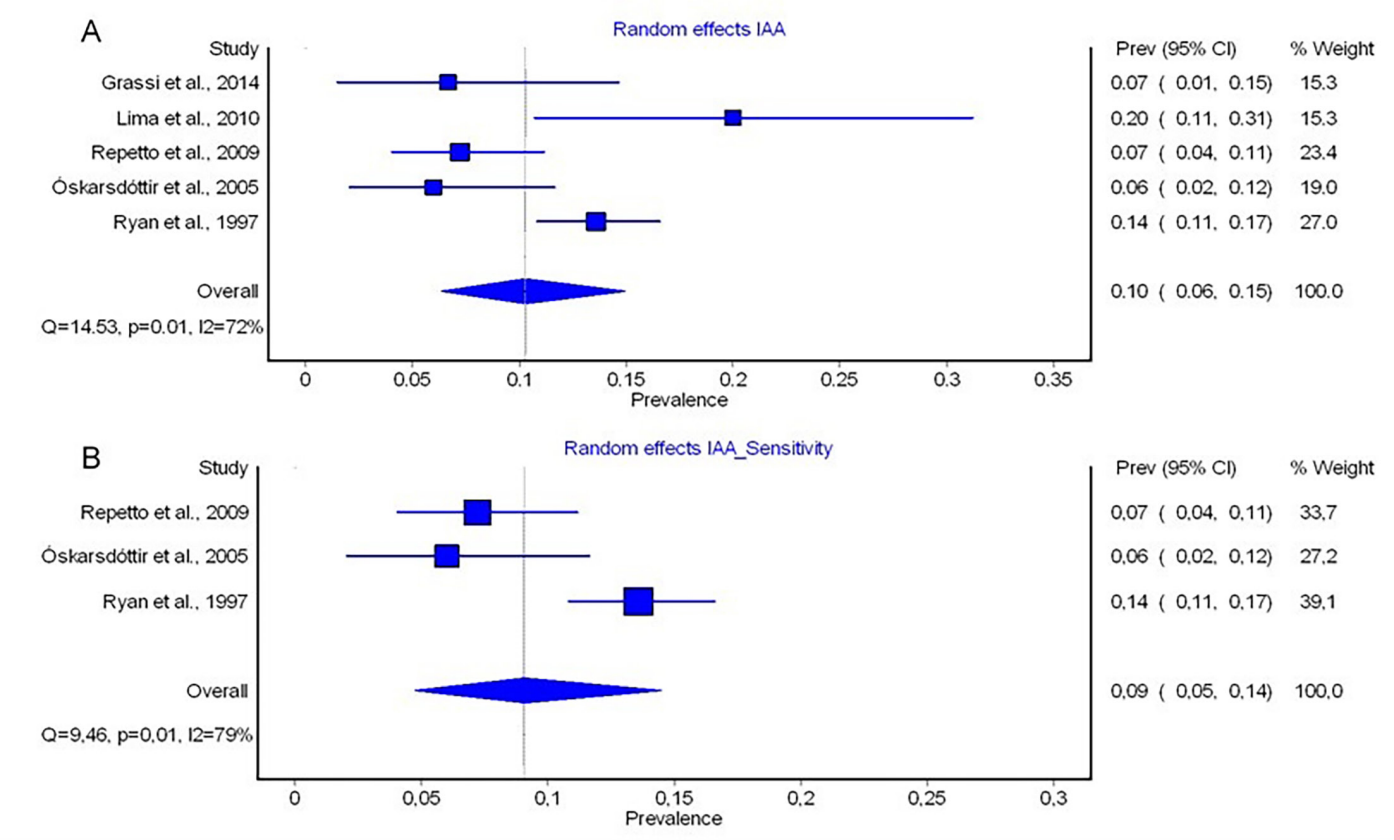
Forest plot of the prevalences of interrupted aortic arch (IAA) calculated by random-effects models in MetaXL (A) and forest plot of the prevalences of IAA calculated by sensitivity analysis by use of random-effects models in MetaXL (B). The included studies are listed on the left side, prevalence rates of each study with 95% CIs are shown on the first column on the right side. The second column on the right side represents the weight a study represents on the meta-analytically calculated pooled prevalence. The diamond on the bottom represents the pooled prevalence. I^2^, percentage of variation in prevalence across studies that is due to heterogeneity rather than chance; p, significance level of Q derived by χ² distribution (df=4); Q, Cochran’s Q.

The prevalence of IAA was reported in five studies. Two studies reported IAA,^22 28^ two other studies reported IAA type B^25 27^ and one study reported IAA+VSD.^24^ There was significant heterogeneity across the five studies (exact Fisher-Freeman-Halton test p=0.005).^22 24 25 27 28^ No outliers were identified via leave-one-out analysis. The pooled prevalence for IAA was 10% (95% CI 0.06%; 0.15%) with a high degree of heterogeneity (I²=72%, Q=14.53, p=0.01)^29^ (figure 5A). A sensitivity analysis, which only included the subset of k=3 studies with a sample size ≥100 individuals, still indicated significant heterogeneity (exact Fisher-Freeman-Halton test p=0.010). The pooled prevalence across these better-powered studies was 9% (95% CI 0.05%; 0.14%), with substantial heterogeneity (I²=79%, Q=9.46, p=0.01) (figure 5B).

## Discussion

To our knowledge, this is the first systematic review and meta-analysis calculating the prevalence of CHD in individuals with 22q11.2DS. Depending on the type of CHD, prevalence rates ranged from 3% to 20%, with TOF showing the highest and ASD showing the lowest.

We observed a prevalence of 14% for VSD and 9% for VSD+PSD in individuals with 22q11.2DS. Compared with those without 22q11.2DS, the prevalence for VSD was 0.262% and 0.307%^10 30^ and for VSD+PSD 0.01%.^31^ This implies that in individuals with 22q11.2DS, the prevalence of VSD may be up to 50-fold higher and of PA+VSD up to 900-fold higher compared with those without. Although the prevalence of VSD and PA+VSD was lower than in narrative literature reviews reported before,^5 12^ we consider our estimates more realistic because those reviews analysed studies that frequently recruited exclusively from paediatric cardiology centres.^32 33^

We found a prevalence of 3% for ASD, which is 20 times higher than the estimates reported by two meta-analyses in patients without 22q11.2DS, that is, 0.164%^30^ and 0.144%,^10^ respectively. Yet, the prevalence of ASD is consistent with data found in the literature review by Momma *et al*, that is, 1–4%.^11^

AAA is frequently associated with CHD in patients with 22q11.2DS.^1127 34^,^36^ However, limited data exist on the prevalence of isolated AAA in both the general population and in those with 22q11.2DS.^37^ Only three studies evaluated the AAA prevalence.^26^,^28^ The relative under-reporting of isolated AAA in the analysed studies^22 24 25^ may be due to the fact that these patients remain asymptomatic for a prolonged period, thus evading early diagnosis. We observed a prevalence range of 2.2–27% for AAA. Since AAA can result from a disturbance of the derivatives of the fourth aortic arch, and since this process appears to be frequent in 22q11.2DS, screening for AAA should be recommended in all infants with 22q11.2DS by a detailed echocardiographic examination.^36 38^ Of note, in adults, such evaluation may require complementary cardiac MRI or CT.^39^

Our meta-analysis found a prevalence of 20% for TOF. As meta-analyses in the general population found a prevalence of ~0.035% for TOF,^10 30^ this suggests that the risk for TOF in individuals with 22q11.2DS may be increased by a factor of almost 600. Due to this strong association, genetic testing for 22q11.2DS in newborn with TOF should be performed. The prevalence of TOF found in this meta-analysis is at the lower boundary compared with the 20–45% prevalence reported in narrative literature reviews.^5 12^ However, as mentioned, many studies entering their database had recruited patients solely from paediatric cardiology centres.^32 33^

The estimated prevalence of TAC (9%) aligns with the findings in several literature reviews.^5 11 12^ TAC is rare in those without 22q11.2DS (0.008%),^10^ but increased by about 1000-fold within the 22q11.2DS population, making it a relevant CHD.

Our data showed a prevalence of 10% for IAA. Whereas it is very rare (0.0041%) in individuals without 22q11.2DS,^10^ IAA is about 2000-fold increased in 22q11.2DS. The studies forming the basis of the here presented meta-analysis reported IAA under the terms ‘IAA’, ‘IAA type B’ and ‘IAA+VSD’. Patients with 22q.11.2DS almost exclusively present with type B,^12 34^ which includes both a conotruncal malformation and a disturbance of the derivatives of the left fourth aortic arch. The predominance of this specific type of IAA is in accordance with the fact that CHD in 22q11.2DS may result from a disturbance of rostral neural crest cells or cells with which these cells interact at a critical phase of organogenesis. The close association of rostral neural crest cells with the formation of the conotruncus and aortic arch derivates explains the increased prevalence of this specific complex CHD in 22q11.2DS.^35 40^

### Limitations and strengths

Although strict inclusion criteria were set, the studies included into the meta-analyses exhibited significant and relevant heterogeneity for some of the outcomes under study (TOF, IAA, TAC). To address this issue, we conducted sensitivity analyses limited to studies with a sample size of ≥100, since it can be assumed that larger studies are more representative.^41^ For TAC, an outlier study^27^ was excluded to arrive at homogeneous groups. For IAA, it was not possible to form non-heterogeneous groups, either by selecting larger studies or by leave-one-out analysis. The studies may have been heterogeneous due to inclusion of different age groups (up to 19.4 years^25^ vs up to 54 years^24^). Recruitment was conducted differently, which could have influenced group constellations and prevalence (see table 1). Additionally, the definition of outcomes differed slightly, including IAA,^22 28^ IAA+VSD^24^ or IAA type B,^25 27^ which could have contributed to heterogeneity. Another limitation is that while the RoB of the individual studies was moderate or low, the overall quality of evidence by GRADE for each outcome was low or very low. This is because only observational studies were found, which by definition are assigned a low level of quality in GRADE. Additionally, due to the dearth of research on 22q11.2DS, the meta-analysis incorporated only a limited number of studies.

### Practical implications and future research

The studies selected for the meta-analyses showed fair selection bias, which lends trust to the results reported here. Consequently, screening for CHD should be conducted in all children with 22q11.2DS,^3^ enabling early treatment strategies to be provided according to guidelines, depending on the specific CHD. Guidelines for the management of 22q11.2DS need to consider including recommendations for cardiological assessment, targeted therapy and long-term cardiological care.^3 42^ Moreover, genetic testing for 22q11.2DS should be included in the diagnostic workup of patients with established CHD so that affected children are identified at an early stage. Practitioners in various medical specialties such as cardiology, psychiatry, otolaryngology or immunology should be informed about the importance of genetic testing for 22q11.2DS, in the presence of typical comorbidities. Genetic counselling providing information regarding aetiology, natural history or recurrence risk should also be offered to affected families.^3^

Some methodological aspects should be considered in conducting prevalence studies. In order to randomly select the population, national birth registers could be implemented where patients with 22q11.2DS, including their comorbidities, are listed. A random selection is also suggested by the Joanna Briggs Institute’s critical appraisal checklist for studies reporting prevalence data.^21^ Additionally, measurement bias should be minimised by using valid and reliable methods of outcome assessment that are comparable across participants.

### Conclusion

We performed a systematic review and meta-analysis on the prevalence of CHD in 22q11.2DS, particularly VSD, PA+VSD, ASD, TOF, TAC and IAA, focusing on studies without selection bias. Given the substantially higher prevalences of CHD in 22q11.2DS compared with general population, screening for CHD is mandatory.

## Supplementary material

10.1136/jmg-2025-110624 online supplemental file 1

## Data Availability

All data generated are available in the present study or online.
