# Supplementary material for: Congenital heart disease in 22q11.2 deletion syndrome: a meta-analysis and systematic review of the literature
Source: J Med Genet. 2025 Jul 31;62(11):e110624. doi: 10.1136/jmg-2025-110624 (PMC12573319; doi:10.1136/jmg-2025-110624)
Supplement: online supplemental file 1 [file jmg-62-11-s001.docx]

**Congenital heart disease in 22q11.2 deletion syndrome: meta-analysis and systematic review of the literature**

Journal of Medical Genetics

Carina Sauter

Department Child and Adolescent Psychiatry, Psychosomatic and Psychotherapy, Center of Mental Health, University Hospital Wuerzburg, Wuerzburg, Germany

Sauter_c@ukw.de

*Full search strategy for each database*

| **Pubmed** | |
| --- | --- |
| Date: 18.08.2022 | |
| Number | Searchterm |
| 1 | 22q11[Title/Abstract] OR deletion 22q11.2[Title/Abstract] OR duplication 22q11.2[Title/Abstract] OR dup22q11[Title/Abstract] OR del22q11[Title/Abstract] OR di-george[Title/Abstract] OR Shprintzen-Goldberg syndrome[Title/Abstract] OR velocardiofacial syndrome[Title/Abstract] OR catch22[Title/Abstract] OR digeorge syndrome[Title/Abstract] |
| 2 | 22q11 deletion syndrome[MeSH Terms] OR digeorge syndrome[MeSH Terms] |
| 3 | #1 OR #2 |
| 4 | cardiovascular[Title/Abstract] OR cardiologic*[Title/Abstract] OR heart defect[Title/Abstract] OR vascular ring[Title/Abstract] OR dilated aortic root[Title/Abstract] OR arrhythmias[Title/Abstract] OR cardiac malformation*[Title/Abstract] |
| 5 | heart defects, congenital[MeSH Terms] OR cardiovascular abnormalities[MeSH Terms] OR arrhythmias, cardiac[MeSH Terms] OR cardiovascular diseases[MeSH Terms] |
| 6 | #4 OR #5 |
| 7 | #3 AND #6 |
| Results: 3587, Update: 22.05.24: 251 new findings | |

| **Cochrane Library** | |
| --- | --- |
| Date: 18.08.2022 | |
| Number | Searchterm |
| 1 | (22q11 OR deletion 22q11.2 OR duplication 22q11.2 OR dup22q11 OR del22q11 OR di-george OR digeorge syndrome OR Shprintzen-Goldberg syndrome OR velocardiofacial syndrome OR catch22):ti,ab,kw |
| 2 | MeSH descriptor: [22q11 Deletion Syndrome] this term only |
| 3 | MeSH descriptor: [DiGeorge Syndrome] this term only |
| 4 | #2 OR #3 |
| 5 | #1 OR #4 (Word variations have been searched) |
| 6 | (cardiovascular OR cardiologic* OR heart defect OR vascular ring OR dilated aortic root OR arrhythmias OR cardiac malformation*):ti,ab,kw |
| 7 | MeSH descriptor: [Heart Defects, Congenital] this term only |
| 8 | MeSH descriptor: [Cardiovascular Abnormalities] this term only |
| 9 | MeSH descriptor: [Arrhythmias, Cardiac] this term only |
| 10 | MeSH descriptor: [Cardiovascular Diseases] this term only |
| 11 | #7 OR #8 OR #9 OR #10 |
| 12 | #6 OR #11 |
| 13 | #5 AND #12 |
| Results: 7, Update: 22.05.24: 0 new findings | |

| **Web of Science** | |
| --- | --- |
| Date: 18.08.2022 | |
| Number | Searchterm |
| 1 | TS=(22q11 OR deletion 22q11.2 OR duplication 22q11.2 OR dup22q11 OR del22q11 OR di-george OR digeorge syndrome OR Shprintzen-Goldberg syndrome OR velocardiofacial syndrome OR catch22) |
| 2 | TS=(cardiovascular OR cardiologic* OR heart defect OR vascular ring OR dilated aortic root OR arrhythmias OR cardiac malformation*) |
| 3 | #1 AND #2 |
| Results: 1366 , Update: 22.05.24: 89 new findings | |

*Evidence tables*

**Tetralogy of Fallot (TOF)**

|  | **Quality assessment** | | | | | **Summary of the results** | | | | **Comment/level of evidence** |
| --- | --- | --- | --- | --- | --- | --- | --- | --- | --- | --- |
| **Study and design** | **Limitations due to the risk of bias** | **Inconsistency** | **Indirectness** | **Lack of precision** | **Publication bias** | **Participants (number and characteristics)** | **Screening method** | **Diagnosis** | **Prevalence** |  |
| Guo et al, 2017 [1]  Retrospective study | According to Hoy et al. low risk of bias (8/10 points) |  |  |  |  | Inclusion of N=1472 individuals with a genetically confirmed 22q11.2 deletion syndrome.  Age: not reported  49% male, 51% female | Echocardiogram and cardiology reports to confirm specific CHD diagnosis | Tetralogy of Fallot | Prevalence of 22%, 95% CI: 0.20; 0.24  (326/1472) | Inclusion in meta-analytic calculation based on sensitivity analysis.  Inclusion of studies n>100: Exact Fisher-Freeman-Halton: .065 not significant🡪 Homogeneous  Weighting: 44% |
| Repetto et al, 2009 [2]  Retrospective study | According to Hoy et al. moderate risk of bias (5/10 points) |  |  |  |  | Inclusion of N=208 individuals with a genetically confirmed 22q11.2 deletion syndrome.  Age: mean 5.2 years (of 182 patients); range from newborn to 39 years  49% male, 51% female | Review of medical records, information on cardiac anatomy using ultrasound, cardiac catheterization or magnetic resonance imaging | Tetralogy of Fallot | Prevalence of 20%, 95% CI: 0.15; 0.26  (42/208) | Inclusion in meta-analytic calculation based on sensitivity analysis.  Inclusion of studies n>100: Exact Fisher-Freeman-Halton: .065 not significant🡪 Homogeneous  Weighting: 21% |
| Ryan et al, 1997 [3]  Retrospective study | According to Hoy et al. moderate risk of bias (6/10 points) |  |  |  |  | Inclusion of N=545 individuals with a genetically confirmed 22q11.2 deletion syndrome.  Age: range from newborn to 51 years old  49% male, 51% female | Data from 23 European centers collected by questionnaires asking for data on heart | Tetralogy of Fallot | Prevalence of 17%, 95% CI: 0.17; 0.23  (96/545) | Inclusion in meta-analytic calculation based on sensitivity analysis.  Inclusion of studies n>100: Exact Fisher-Freeman-Halton: .065 not significant🡪 Homogeneous  Weighting 34% |
| **GRADE rating**  **Study and design** | **Limitations due to risk of bias** | **Inconsistency** | **Indirectness** | **Lack of precision** | **Publication bias** | **Final evidence assessment on the endpoint "prevalence of Tetralogy of Fallot".** | **Result of the meta-analytical calculation** | | | |
| Output:  ⊕⊕ | Moderate risk of bias | None | None | None | Not assessable | Evidence assessment for ⊕⊕-low-quality studies. | Pooled prevalence (random-effects model): 20%, 95% CI: 0.17; 0.23 | | | |

| Studies that were not included in the meta-analytic calculations because significance according to the Fisher-Freeman-Halton: <.001 was given (significant🡪 inhomogeneous). | | | | | | | | | | |
| --- | --- | --- | --- | --- | --- | --- | --- | --- | --- | --- |
| Grassi et al, 2014 [4]  Cross-sectional study | According to Hoy et al. moderate risk of bias (6/10 points) |  |  |  |  | Inclusion of N=60 individuals with a genetically confirmed 22q11.2 deletion syndrome.  Age: mean 4.8 years, range from 14 days to 20 years  57% male, 43% female | clinical and imaging examinations in the pediatric cardiology department | Tetralogy of Fallot | Prevalence of 30%, 95% CI: 0.19, 0.42  (18/60) | - |
| Lima et al, 2010 [5]  Cross-sectional study | According to Hoy et al. low risk of bias (8/10 points) |  |  |  |  | Inclusion of N=60 individuals with a genetically confirmed 22q11.2 deletion syndrome.  Age: range from 1 to 54 years  47% male, 53% female | Comparison of data obtained in an interview with patients' medical records, drawing on reports of echocardiograms, cardiac catheterizations, and radiographs | Tetralogy of Fallot | Prevalence of 7%, 95% CI: 0.01; 0.15  (4/60) | - |
| Óskarsdóttir et al, 2005. [6]  Cross-sectional study | According to Hoy et al. low risk of bias (9/10 points) |  |  |  |  | Inclusion of N=100 individuals with a genetically confirmed 22q11.2 deletion syndrome.  Age: range from 0.01 to 19.4 years  46% male, 54% female | Review of medical records, information on cardiac anatomy using ultrasound, cardiac catheterization or magnetic resonance imaging | Tetralogy of Fallot | Prevalence of 13%, 95% CI: 0.07 0.20  (13/100) | - |

CHD: congenital heart disease; CI: confidence interval; TOF: Tetralogy of Fallot

Fig.S1 Forest plot of the pooled prevalence of tetralogy of Fallot (TOF) calculated of all studies by random-effects models in MetaXL. Groups were not homogeneous.

Fig.S2 Forest plot of the pooled prevalence of tetralogy of Fallot (TOF) calculated by three studies with n>100 by random-effects models in MetaXL.

**Pulmonary atresia with ventricular septal defect (PA + VSD)**

|  | **Quality assessment** | | | | | **Summary of the results** | | | | **Comment/level of evidence** |
| --- | --- | --- | --- | --- | --- | --- | --- | --- | --- | --- |
| **Study and design** | **Limitations due to the risk of bias** | **Inconsistency** | **Indirectness** | **Lack of precision** | **Publication bias** | **Participants (number and characteristics)** | **Screening method** | **Diagnosis** | **Prevalence** |  |
| Lima et al, 2010 [5]  Cross-sectional study | According to Hoy et al. low risk of bias (8/10 points) |  |  |  |  | Inclusion of N=60 individuals with a genetically confirmed 22q11.2 deletion syndrome.  Age: range from 1 to 54 years  47% male, 53% female | Comparison of data obtained in an interview with patients' medical records, drawing on reports of echocardiograms, cardiac catheterizations, and radiographs. | Pulmonary atresia with ventricular septal defect | Prevalence of 10%, 95% CI: 0.03; 0.19  (6/60) | Inclusion in the meta-analytic calculation  Exact Fisher-Freeman-Halton: = .287 not significant🡪 Homogeneous  Weighting 15% |
| Óskarsdóttir et al, 2005 [6]  Cross-sectional study | According to Hoy et al. low risk of bias (9/10 points) |  |  |  |  | Inclusion of N=100 individuals with a genetically confirmed 22q11.2 deletion syndrome.  Age: range from 0.01 to 19.4 years  46% male, 54% female | Review of medical records, information on cardiac anatomy using ultrasound, cardiac catheterization or magnetic resonance imaging. | Pulmonary atresia with ventricular septal defect | Prevalence of 5%, 95% CI: 0.01; 0.10  (5/100) | Inclusion in the meta-analytic calculation  Exact- Fisher-Freeman-Halton: =.287 not significant🡪 Homogeneous  Weighting 23% |
| Ryan et al, 1997 [3]  Retrospective study | According to Hoy et al. moderate risk of bias (6/10 points) |  |  |  |  | Inclusion of N=545 individuals with a genetically confirmed 22q11.2 deletion syndrome.  Age: range from newborn to 51 years old  49% male, 51% female | Data from 23 European centers collected by questionnaires asking for data on heart | Pulmonary atresia with ventricular septal defect | Prevalence of 10%, 95% CI: 0.08; 0.13  (55/545) | Inclusion in the meta-analytic calculation  Exact- Fisher-Freeman-Halton: =.287 not significant🡪 Homogeneous  Weighting 62% |
| **GRADE rating**  **Study and design** | **Limitations due to the risk of bias** | **Inconsistency** | **Indirectness** | **Lack of precision** | **Publication bias** | **Final evidence assessment on the endpoint "prevalence of pulmonary atresia with ventricular septal defect."** | **Result of the meta-analytical calculation** | | | |
| Output:  ⊕⊕ | Low to moderate risk of bias | None | None | None | Not assessable | Evidence assessment for ⊕⊕-low-quality studies | Pooled prevalence (random-effects model): 9%, 95% CI: 0.06; 0.12 | | | |

CI: confidence interval; PA + VSD: Pulmonary atresia with ventricular septal defect

Fig.S3 Forest plot of the pooled prevalence of pulmonary atresia and ventricular septal defect (PA +VSD) calculated of all studies by random-effects models in MetaXL.

**Truncus arteriosus communis (TAC)**

|  | **Quality assessment** | | | | | **Summary of the results** | | | | **Comment/level of evidence** |
| --- | --- | --- | --- | --- | --- | --- | --- | --- | --- | --- |
| **Study and design** | **Limitations due to the risk of bias** | **Inconsistency** | **Indirectness** | **Lack of precision** | **Publication bias** | **Participants (number and characteristics)** | **Screening method** | **Diagnosis** | **Prevalence** |  |
| Grassi et al, 2014 [4]  Cross-sectional study | According to Hoy et al. moderate risk of bias (6/10 points) |  |  |  |  | Inclusion of N=60 individuals with a genetically confirmed 22q11.2 deletion syndrome.  Age: mean 4.8 years, range from 14 days to 20 years  57% male, 43% female | Clinical and imaging examinations in the pediatric cardiology department | Truncus arteriosus communis | Prevalence of 7%, 95% CI: 0.01; 0.15  (4/60) | Inclusion in meta-analytic calculation based on sensitivity analysis.  Exact Fisher-Freeman-Halton: .823 not significant🡪 Homogeneous  Weighting 9% |
| Óskarsdóttir et al, 2005. [6]  Cross-sectional study | According to Hoy et al. low risk of bias (9/10 points) |  |  |  |  | Inclusion of N=100 individuals with a genetically confirmed 22q11.2 deletion syndrome.  Age: range from 0.01 to 19.4 years  46% male, 54% female | Review of medical records, information on cardiac anatomy using ultrasound, cardiac catheterization or magnetic resonance imaging. | Truncus arteriosus communis | Prevalence of 10%, 95% CI: 0.05; 0.17  (10/100) | Inclusion in meta-analytic calculation based on sensitivity analysis.  Exact Fisher-Freeman-Halton: .823 not significant🡪 Homogeneous  Weighting 14% |
| Ryan et al, 1997 [3]  Retrospective study | According to Hoy et al. moderate risk of bias (6/10 points) |  |  |  |  | Inclusion of N=545 individuals with a genetically confirmed 22q11.2 deletion syndrome.  Age: range from newborn to 51 years old  49% male, 51% female | Data from 23 European centers collected by questionnaires asking for data on heart | Truncus arteriosus communis | Prevalence of 9%, 95% CI: 0.07; 0.12  (51/545) | Inclusion in meta-analytic calculation based on sensitivity analysis.  Exact Fisher-Freeman-Halton: .823 not significant🡪 Homogeneous  Weighting 77% |
| **GRADE rating**  **Study and design** | **Limitations due to risk of bias** | **Inconsistency** | **Indirectness** | **Lack of precision** | **Publication bias** | **Final evidence assessment on the endpoint "prevalence of truncus arteriosus communis".** | **Result of the meta-analytical calculation** | | | |
| Output:  ⊕⊕ | Moderate risk of bias | None | None | None | Not assessable | Evidence assessment for ⊕⊕-low-quality studies. | Pooled prevalence (random-effects model): 9%, 95% CI: 0.07; 0.12 | | | |

| Studies that were not included in the meta-analytical calculations, because significance according to the Fisher-Freeman-Halton: .004 was given (significant🡪 inhomogeneous). | | | | | | | | | | |
| --- | --- | --- | --- | --- | --- | --- | --- | --- | --- | --- |
| Repetto et al, 2009 [2]  Retrospective study | According to Hoy et al. moderate risk of bias (5/10 points) |  |  |  |  | Inclusion of N=208 individuals with a genetically confirmed 22q11.2 deletion syndrome.  Age: mean: 5.2 years (of 182 patients); range from newborn to 39 years  49% male, 51% female | Review of medical records, information on cardiac anatomy using ultrasound, cardiac catheterization or magnetic resonance imaging | Truncus arteriosus communis | Prevalence of 2%, 95% CI: 0.01; 0.05  (5/208) |  |

CI: confidence interval; TAC: truncus arteriosus communis

Fig.S4 Forest plot of the pooled prevalence of truncus arteriosus communis (TAC) calculated of all studies by random-effects models in MetaXL. Groups were not homogeneous.

Fig.S5 Forest plot of the pooled prevalence of truncus arteriosus communis (TAC) calculated without an outlier study by random-effects models in MetaXL.

**Interrupted aortic arch (IAA)**

|  | **Quality assessment** | | | | | **Summary of the results** | | | | **Comment/level of evidence** |
| --- | --- | --- | --- | --- | --- | --- | --- | --- | --- | --- |
| **Study and design** | **Limitations due to risk of bias** | **Inconsistency** | **Indirectness** | **Lack of precision** | **Publication bias** | **Participants (number and characteristics)** | **Screening method** | **Diagnosis** | **Prevalence** |  |
| Grassi et al, 2014 [4]  Cross-sectional study | According to Hoy et al. moderate risk of bias (6/10 points) |  |  |  |  | Inclusion of N=60 individuals with a genetically confirmed 22q11.2 deletion syndrome.  Age: mean 4.8 years, range from 14 days to 20 years  57% male, 43% female | Clinical and imaging examinations in the pediatric cardiology department | Interrupted aortic arch | Prevalence of 7%, 95% CI: 0.01, 0.15  (4/60) | Inclusion in the meta-analytic calculation  All studies: Exact Fisher-Freeman-Halton: .005 significant🡪 Inhomogeneous  Weighting: 16% |
| Lima et al, 2010 [5]  Cross-sectional study | According to Hoy et al. low risk of bias (8/10 points) |  |  |  |  | Inclusion of N=60 individuals with a genetically confirmed 22q11.2 deletion syndrome.  Age: range from 1 to 54 years  47% male, 53% female | Comparison of data obtained in an interview with patients' medical records, drawing on reports of echocardiograms, cardiac catheterizations, and radiographs | Interrupted aortic arch and ventricular septal defect | Prevalence of 20%, 95% CI: 0.11; 0.31  (12/60) | Inclusion in the meta-analytic calculation  All studies: Exact Fisher-Freeman-Halton: .005 significant🡪 Inhomogeneous  Weighting: 15% |
| Repetto et al, 2009 [2]  Retrospective study | According to Hoy et al. moderate risk of bias (5/10 points) |  |  |  |  | Inclusion of N=208 individuals with a genetically confirmed 22q11.2 deletion syndrome.  Age: mean 5.2 years (of 182 patients); range from newborn to 39 years  49% male, 51% female | Review of medical records, information on cardiac anatomy using ultrasound, cardiac catheterization or magnetic resonance imaging | Interrupted aortic arch Type B | Prevalence of 7%, 95% CI: 0.04; 0.11  (15/208) | Inclusion in the meta-analytic calculation  All studies: Exact Fisher-Freeman-Halton: .005 significant🡪 Inhomogeneous  Weighting: 23% |
| Óskarsdóttir et al, 2005. [6]  Cross-sectional study | According to Hoy et al. low risk of bias (9/10 points) |  |  |  |  | Inclusion of N=100 individuals with a genetically confirmed 22q11.2 deletion syndrome.  Age: range from 0.01 to 19.4 years  46% male, 54% female | Review of medical records, information on cardiac anatomy using ultrasound, cardiac catheterization or magnetic resonance imaging | Interrupted aortic arch Type B | Prevalence of 6%, 95% CI: 0.02, 0.12  (6/100) | Inclusion in the meta-analytic calculation  All studies: Exact Fisher-Freeman-Halton: .005 significant🡪 Inhomogeneous  Weighting: 19% |
| Ryan et al, 1997 [3]  Retrospective study | According to Hoy et al. moderate risk of bias (6/10 points) |  |  |  |  | Inclusion of N=545 individuals with a genetically confirmed 22q11.2 deletion syndrome.  Age: range from newborn to 51 years old  49% male, 51% female | Data from 23 European centers collected by questionnaires asking for data on heart | Interrupted aortic arch | Prevalence of 14%, 95% CI: 0.11; 0.17  (74/545) | Inclusion in the meta-analytic calculation  All studies: Exact Fisher-Freeman-Halton: .005 significant🡪 Inhomogeneous  Weighting: 27% |
| **GRADE rating**  **Study and design** | **Limitations due to the risk of bias** | **Inconsistency** | **Indirectness** | **Lack of precision** | **Publication bias** | **Final evidence assessment on the endpoint "interrupted aortic arch".** | **Result of the meta-analytical calculation** | | | |
| Output:  ⊕⊕ | Moderate risk of bias | Given, as different prevalence range, High heterogeneity  -1 point regarding quality according to GRADE | None | None | Not assessable | Evidence assessment at ⊕ -very low study quality. | Pooled prevalence (random-effects model): 10%, 95% CI: 0.06; 0.15  However, high heterogeneity | | | |

CI: confidence interval; IAA: Interrupted aortic arch

Fig.S6 Forest plot of the pooled prevalence of interrupted aortic arch (IAA) calculated of all studies by random-effects models in MetaXL. Substantial heterogeneity given.

Fig.S2 Forest plot of the pooled prevalence of interrupted aortic arch (IAA) calculated by three studies with n>100 by random-effects models in MetaXL.

**Ventricular septal defect (VSD)**

|  | **Quality assessment** | | | | | **Summary of the results** | | | | **Comment/level of evidence** |
| --- | --- | --- | --- | --- | --- | --- | --- | --- | --- | --- |
| **Study and design** | **Limitations due to the risk of bias** | **Inconsistency** | **Indirectness** | **Lack of precision** | **Publication bias** | **Participants (number and characteristics)** | **Screening method** | **Diagnosis** | **Prevalence** |  |
| Grassi et al, 2014 [4]  Cross-sectional study | According to Hoy et al. moderate risk of bias (6/10 points) |  |  |  |  | Inclusion of N=60 individuals with a genetically confirmed 22q11.2 deletion syndrome.  Age: mean 4.8 years, range from 14 days to 20 years  57% male, 43% female | clinical and imaging examinations in the pediatric cardiology department | Ventricular septal defect | Prevalence of 17%, 95% CI: 0.08, 0.27  (10/60) | Inclusion in meta-analytic calculation, Exact Fisher-Freeman-Halton: .401 not significant🡪 Homogeneous  Weighting: 6% |
| Lima et al, 2010 [5]  Cross-sectional study | According to Hoy et al. low risk of bias (8/10 points) |  |  |  |  | Inclusion of N=60 individuals with a genetically confirmed 22q11.2 deletion syndrome.  Age: range from 1 to 54 years  47% male, 53% female | Comparison of data obtained in an interview with patients' medical records, drawing on reports of echocardiograms, cardiac catheterizations, and radiographs | Ventricular septal defect | Prevalence of 22%, 95% CI: 0.12; 0.33  (13/60) | Inclusion in meta-analytic calculation, Exact Fisher-Freeman-Halton: .401 not significant🡪 Homogeneous  Weighting: 6% |
| Repetto et al, 2009 [2]  Retrospective study | According to Hoy et al. moderate risk of bias (5/10 points) |  |  |  |  | Inclusion of N=208 individuals with a genetically confirmed 22q11.2 deletion syndrome.  Age: mean: 5.2 years (of 182 patients); range from newborn to 39 years  49% male, 51% female | Review of medical records, information on cardiac anatomy using ultrasound, cardiac catheterization or magnetic resonance imaging | Ventricular septal defect | Prevalence of 12%, 95% CI: 0.08; 0.17  (25/208) | Inclusion in meta-analytic calculation, Exact Fisher-Freeman-Halton: .401 not significant🡪 Homogeneous  Weighting: 21% |
| Óskarsdóttir et al, 2005. [6]  Cross-sectional study | According to Hoy et al. low risk of bias (9/10 points) |  |  |  |  | Inclusion of N=100 individuals with a genetically confirmed 22q11.2 deletion syndrome.  Age: range from 0.01 to 19.4 years  46% male, 54% female | Review of medical records, information on cardiac anatomy using ultrasound, cardiac catheterization or magnetic resonance imaging | Ventricular septal defect | Prevalence of 14%, 95% CI: 0.08, 0.22  (14/100) | Inclusion in meta-analytic calculation, Exact Fisher-Freeman-Halton: .401 not significant🡪 Homogeneous  Weighting: 10% |
| Ryan et al, 1997 [3]  Retrospective study | According to Hoy et al. moderate risk of bias (6/10 points) |  |  |  |  | Inclusion of N=545 individuals with a genetically confirmed 22q11.2 deletion syndrome.  Age: range from newborn to 51 years old  49% male, 51% female | Data from 23 European centers collected by questionnaires asking for data on heart | Ventricular septal defect | Prevalence of 14%, 95% CI: 0.11; 0.16  (75/545) | Inclusion in meta-analytic calculation, Exact Fisher-Freeman-Halton: .401 not significant🡪 Homogeneous  Weighting: 56% |
| **GRADE rating**  **Study and design** | **Limitations due to the risk of bias** | **Inconsistency** | **Indirectness** | **Lack of precision** | **Publication bias** | **Final Evidence Assessment on the Endpoint "Ventricular Septal Defect "** | **Result of the meta-analytical calculation** | | | |
| Output:  ⊕⊕ | Moderate risk of bias | None | None | None | Not assessable | Evidence assessment for ⊕⊕-low-quality studies | Pooled prevalence (random-effects model): 14%, 95% CI: 0.12; 0.16 | | | |

CI: confidence interval; VSD: Ventricular septal defect

Fig.S7 Forest plot of the pooled prevalence of ventricular septal defect (VSD) calculated of all studies by random-effects models in MetaXL.

**Atrial septal defect (ASD)**

|  | **Quality assessment** | | | | | **Summary of the results** | | | | **Comment/level of evidence** |
| --- | --- | --- | --- | --- | --- | --- | --- | --- | --- | --- |
| **Study and design** | **Limitations due to the risk of bias** | **Inconsistency** | **Indirectness** | **Lack of precision** | **Publication bias** | **Participants (number and characteristics)** | **Screening method** | **Diagnosis** | **Prevalence** |  |
| Grassi et al, 2014 [4]  Cross-sectional study | According to Hoy et al. moderate risk of bias (6/10 points) |  |  |  |  | Inclusion of N=60 individuals with a genetically confirmed 22q11.2 deletion syndrome.  Age: average: 4.8 years, range from 14 days to 20 years  57% male, 43% female | Clinical and imaging examinations in the pediatric cardiology department | Atrial septal defect | Prevalence of 5%, 95% CI: 0.01, 0.12  (3/60) | Inclusion in meta-analytic calculation, Exact Fisher-Freeman-Halton: .060 not significant🡪 Homogeneous  Weighting: 11% |
| Lima et al, 2010 [5]  Cross-sectional study | According to Hoy et al. low risk of bias (8/10 points) |  |  |  |  | Inclusion of N=60 individuals with a genetically confirmed 22q11.2 deletion syndrome.  Age: range from 1 to 54 years  47% male, 53% female | Comparison of data obtained in an interview with patients' medical records, drawing on reports of echocardiograms, cardiac catheterizations, and radiographs | Atrial septal defect | Prevalence of 5%, 95% CI: 0.01; 0.12  (3/60) | Inclusion in meta-analytic calculation, Exact Fisher-Freeman-Halton: .060 not significant🡪 Homogeneous  Weighting: 11% |
| Repetto et al, 2009 [2]  Retrospective study | According to Hoy et al. moderate risk of bias (5/10 points). |  |  |  |  | Inclusion of N=208 individuals with a genetically confirmed 22q11.2 deletion syndrome.  Age: mean 5.2 years (of 182 patients); range from newborn to 39 years  49% male, 51% female | Review of medical records, information on cardiac anatomy using ultrasound, cardiac catheterization or magnetic resonance imaging. | Atrial septal defect | Prevalence of 3%, 95% CI: 0.01; 0.06  (6/208) | Inclusion in meta-analytic calculation, Exact Fisher-Freeman-Halton: .060 not significant🡪 Homogeneous  Weighting: 25% |
| Óskarsdóttir et al, 2005. [6]  Cross-sectional study | According to Hoy et al. low risk of bias (9/10 points). |  |  |  |  | Inclusion of N=100 individuals with a genetically confirmed 22q11.2 deletion syndrome.  Age: range from 0.01 to 19.4 years  46% male, 54% female | Review of medical records, information on cardiac anatomy using ultrasound, cardiac catheterization or magnetic resonance imaging | Atrial septal defect | Prevalence of 1%, 95% CI: 0.00, 0.04  (1/100) | Inclusion in meta-analytic calculation, Exact Fisher-Freeman-Halton: .060 not significant🡪 Homogeneous  Weighting: 16% |
| Ryan et al, 1997 [3]  Retrospective study | According to Hoy et al. moderate risk of bias (6/10 points) |  |  |  |  | Inclusion of N=545 individuals with a genetically confirmed 22q11.2 deletion syndrome.  Age: range from newborn to 51 years old  49% male, 51% female | Data from 23 European centers collected by questionnaires asking for data on heart | Atrial septal defect | Prevalence of 1%, 95% CI: 0.01; 0.03  (8/545) | Inclusion in meta-analytic calculation, Exact Fisher-Freeman-Halton: .060 not significant🡪 Homogeneous  Weighting: 38% |
| **GRADE rating**  **Study and design** | **Limitations due to the risk of bias** | **Inconsistency** | **Indirectness** | **Lack of precision** | **Publication bias** | **Final evidence assessment on the endpoint "atrial septal defect ".** | **Result of the meta-analytical calculation** | | | |
| Output:  ⊕⊕ | Moderate risk of bias | None | None | None | Not assessable | Evidence assessment for ⊕⊕-low-quality studies | Pooled prevalence (random-effects model): 3%, 95% CI: 0.01; 0.04 | | | |

CI: confidence interval; ASD: Atrial septal defect

Fig.S8 Forest plot of the pooled prevalence of atrial septal defect (ASD) calculated of all studies by random-effects models in MetaXL.

**Aortic arch anomalies (AAA)**

|  | **Quality assessment** | | | | | **Summary of the results** | | | | **Comment/level of evidence** |
| --- | --- | --- | --- | --- | --- | --- | --- | --- | --- | --- |
| **Study and design** | **Limitations due to the risk of bias** | **Inconsistency** | **Indirectness** | **Lack of precision** | **Publication bias** | **Participants (number and characteristics)** | **Screening method** | **Diagnosis** | **Prevalence** |  |
| Ryan et al, 1997 [3]  Retrospective study | According to Hoy et al. moderate risk of bias (6/10 points) |  |  |  |  | Inclusion of N=545 individuals with a genetically confirmed 22q11.2 deletion syndrome.  Age: range from newborn to 51 years old  49% male, 51% female | Data from 23 European centers collected by questionnaires asking for data on heart | Right aortic arch (n=5), aberrant subclavian artery (n=5), double aortic arch (n=2) | Prevalence of 2.2%, 95% CI: 0.01; 0.04  (12/545) |  |
| Repetto et al, 2009 [2]  Retrospective study | According to Hoy et al. moderate risk of bias (5/10 points) |  |  |  |  | Inclusion of N=208 individuals with a genetically confirmed 22q11.2 deletion syndrome.  Age: mean 5.2 years (of 182 patients); range from newborn to 39 years  49% male, 51% female | Review of medical records, information on cardiac anatomy using ultrasound, cardiac catheterization or magnetic resonance imaging. | Other anomalies including AAA (aberrant subclavian artery, right sided aortic arch, vascular ring and double aortic arch but also patent ductus arteriosus | Prevalence of 13% (2//208) |  |
| Putotto et al., 2022 [7]  Longitudinal single-center study | According to Hoy et al. low risk of bias (8/10 points) |  |  |  |  | Inclusion of N=74 individuals with a genetically confirmed 22q11.2 deletion syndrome.  Age: mean 27,5 years; ≥16 years  59,5% male, 40,5% female | Transthoracic echocardiogramm | Aortic arch/epiaortic vessel anomalies (double aortic arch (n=1), right aortic arch (n=15), right aortic arch with aberrant left subclavian artery (n=8), left aortic arch with aberrant right subclavian artery (n=5), kommerell diverticulum (n=3), vascular ring (n=2)) | Prevalence of 27.0%, 95% CI: 0.17; 0.38 (20/74) |  |

| **GRADE rating**  **Study and design** | **Limitations due to the risk of bias** | **Inconsistency** | **Indirectness** | **Lack of precision** | **Publication bias** | **Final evidence assessment on the endpoint "aortic arch anomalies "** | **Result of the meta-analytical calculation** |
| --- | --- | --- | --- | --- | --- | --- | --- |
| Output:  ⊕⊕ | Moderate to low risk of bias | None | None | No clear description of the outcomes  -1 point regarding study quality according to GRADE | Not assessable | Evidence assessment at ⊕ -very low study quality. | See above |

CI: confidence interval; AAA: Aortic arch anomalies


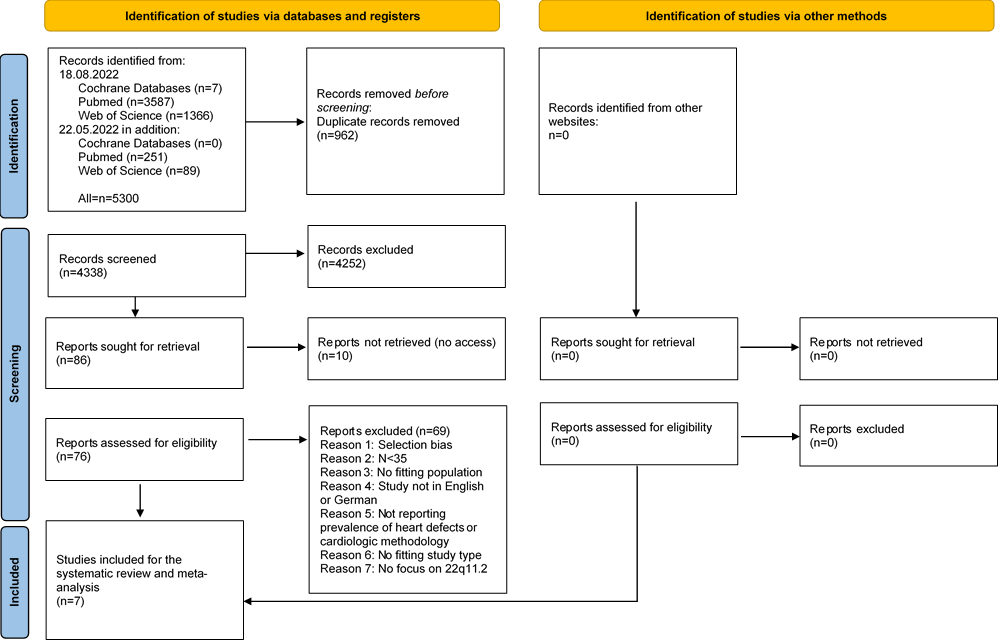


Fig.S9 PRISMA flow chart showing the systematic literature search

REFERENCES

1 Guo TW, Repetto GM, McGinn DMM, et al. Genome-Wide Association Study to Find Modifiers for Tetralogy of Fallot in the 22q11.2 Deletion Syndrome Identifies Variants in the &ITGPR98&IT Locus on 5q14.3. *Circ Cardiovasc Genet.* 2017;10(5).

2 Repetto GM, Guzman ML, Puga A, et al. Clinical features of chromosome 22q11.2 microdeletion syndrome in 208 Chilean patients. *Clinical Genetics* 2009;76(5):465–70.

3 Ryan AK, Goodship JA, Wilson DI, et al. Spectrum of clinical features associated with interstitial chromosome 22q11 deletions: a European collaborative study. *J Med Genet* 1997;34(10):798–804.

4 Grassi MS, Jacob CMA, Kulikowski LD, et al. Congenital Heart Disease as a Warning Sign for the Diagnosis of the 22q11.2 Deletion. *Arquivos Brasileiros De Cardiologia* 2014;103(5):382–90.

5 Lima K, Følling I, Eiklid KL, et al. Age-dependent clinical problems in a Norwegian national survey of patients with the 22q11.2 deletion syndrome. *Eur J Pediatr* 2010;169(8):983–89. doi:10.1007/s00431-010-1161-3 [published Online First: 20100226].

6 Óskarsdóttir S, Persson C, Eriksson BO, et al. Presenting phenotype in 100 children with the 22q11 deletion syndrome. *Eur J Pediatr* 2005;164(3):146–53. doi:10.1007/s00431-004-1577-8 [published Online First: 20041123].

7 Putotto C, Pulvirenti F, Pugnaloni F, et al. Clinical Risk Factors for Aortic Root Dilation in Patients with 22q11.2 Deletion Syndrome: A Longitudinal Single-Center Study. *Genes* 2022;13(12). doi:10.3390/genes13122334 [published Online First: 10 December 2022].
